# OutCyte: a novel tool for predicting unconventional protein secretion

**DOI:** 10.1038/s41598-019-55351-z

**Published:** 2019-12-19

**Authors:** Linlin Zhao, Gereon Poschmann, Daniel Waldera-Lupa, Nima Rafiee, Markus Kollmann, Kai Stühler

**Affiliations:** 1 Institute of Molecular Medicine, Medical Faculty, Heinrich-Heine-University, Düsseldorf, Germany; 2 Mathematical Modelling of Biological Systems, Heinrich-Heine-University, Düsseldorf, Germany; 3 Molecular Proteomics Laboratory, BMFZ, Heinrich-Heine-University, Düsseldorf, Germany

**Keywords:** Protein function predictions, Software

## Abstract

The prediction of protein localization, such as in the extracellular space, from high-throughput data is essential for functional downstream inference. It is well accepted that some secreted proteins go through the classic endoplasmic reticulum-Golgi pathway with the guidance of a signal peptide. However, a large number of proteins have been found to reach the extracellular space by following unconventional secretory pathways. There remains a demand for reliable prediction of unconventional protein secretion (UPS). Here, we present OutCyte, a fast and accurate tool for the prediction of UPS, which for the first time has been built upon experimentally determined UPS proteins. OutCyte mediates the prediction of protein secretion in two steps: first, proteins with N-terminal signals are accurately filtered out; second, proteins without N-terminal signals are classified as UPS or intracellular proteins based on physicochemical features directly generated from their amino acid sequences. We are convinced that OutCyte will play a relevant role in the annotation of experimental data and will therefore contribute to further characterization of the extracellular nature of proteins by considering the commonly neglected UPS proteins.

*OutCyte has been implemented as a web server at* www.outcyte.com.

## Introduction

A protein’s identity is determined by not only its structure but also its cellular localization, which is associated with specific post-translational modification patterns as well as interaction partners. Therefore, the determination of protein localization by experimental approaches or prediction tools is a relevant task for annotating complex data sets obtained from global approaches such as genomics, transcriptomics or proteomics, allowing novel functional inferences. Among proteins transported into the extracellular space, reliable prediction is already well established for proteins with an N-terminal signal peptide^1,2^. The so-called classic protein secretion is a signal-based process that occurs via the endoplasmic reticulum (ER)-Golgi route and is highly conserved in yeast, plant and animal cells^3^. Secretory pathways that act without the involvement of an identified N-terminal signal peptide are classified under the term “unconventional protein secretion” (UPS)^4–6^. The lack of an identified clear sequence patterns for UPS) and the low number of known UPS proteins have thus far been major obstacles for the generation of meaningful prediction tools. The existing computational tools for predicting UPS, such as SecretomeP^7^ and SPRED^8^, make use of classic secretory proteins by removing their signal peptides, based on the hypothesis that all secretory proteins share common features regardless of the specific pathways.

Here, we introduce OutCyte, an integrated tool featuring two modules for predicting unconventional secreted proteins in eukaryotes. In contrast to the existing tools, the module for predicting potential UPS (OutCyte-UPS) was built, on one hand, with our in-house experimental secretome data sets, using features directly generated from protein sequences. On the other hand, we decided to filter these proteins by using an addition module (OutCyte-SP) to avoid interference from other secreted proteins with reliable predictable N-terminal signal peptides (classic secretion, ectodomain shedding of membrane proteins^9^). OutCyte-SP is based on convolutional neural networks that allow the filtering of proteins with N-terminal signals before application of OutCyte-UPS for the prediction of UPS proteins.

## Results and Discussion

**Figure 1:**
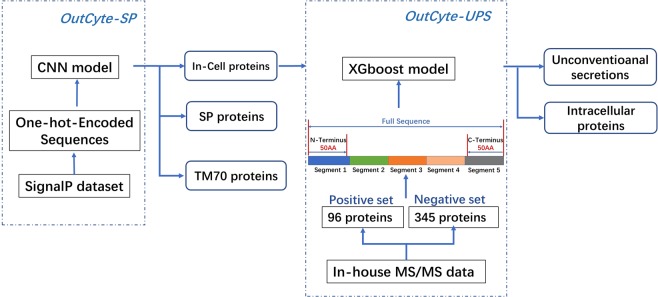
The OutCyte framework is an integrated predictive tool for signal peptide-containing proteins and unconventionally secreted proteins. OutCyte-SP classifies input proteins into three categories: proteins with a signal peptide, proteins with a transmembrane domain at the N-terminus, or proteins not belonging to these two classes. The latter proteins were further analysed by OutCyte-UPS, which has been trained on experimentally determined secreted proteins and classifies input proteins as intracellular or unconventionally secreted.

OutCyte has been designed in a modular fashion (Fig. 1) and includes two modules: OutCyte-SP classifies proteins exhibiting a signal peptide or transmembrane domain within the first 70 amino acids. Thus, we are able to independently determine N-terminal signal peptides or transmembrane domains. The group of proteins without such sequence motifs are fed into OutCyte-UPS for predicting UPS proteins from this group. OutCyte differs from other tools developed for predicting UPS in two main aspects. First, instead of relying on database information that might be prone to false positive results, OutCyte has been trained on experimental data from secretome analysis of several cell lines. Second, OutCyte provides two layers of prediction to avoid false positive UPS prediction arising from proteins containing signal peptides or transmembrane domains at the N-terminus.

### OutCyte-SP: prediction of N-terminal signals

**Figure 2:**
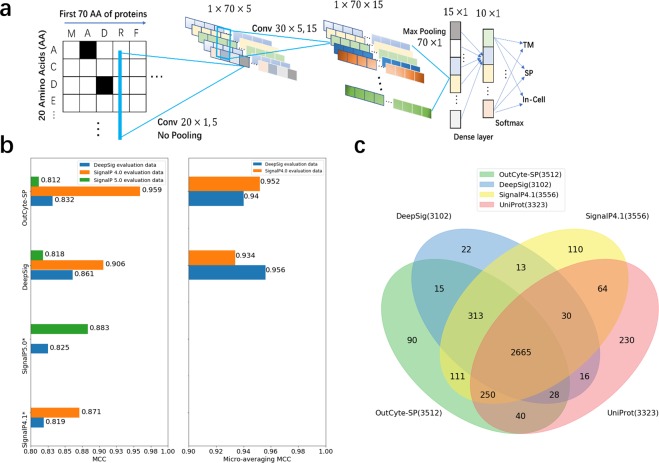
The OutCyte-SP model and its predictions. (**a**) The structure of the convolutional neural network for learning the motifs at the N-termini of sequences. The network consists of two convolutional layers, which use ReLu transformations and no pooling. A max pooling layer follows to extract the strongest distinguishing features, followed by the dense and softmax layers. (**b**) Matthews correlation coefficients (MCCs) for signal peptide identification from three datasets are shown in the left panel. In the right panel, micro-averaged MCCs were calculated for OutCyte-SP and DeepSig on the two evaluation datasets. *The SignalP5.0 training dataset overlapped with SignalP4.0’s benchmark set; thus, two MCCs were not included. (**c**) Intersections among 4 different annotations for signal-peptide-containing proteins in the human proteome from OutCyte-SP, UniProt (with evidence), SignalP 4.1 and DeepSig.

OutCyte-SP annotates proteins with an N-terminal signal peptide or transmembrane domain (Fig. 1). Here, we trained a convolutional neural network model (CNN) with a novel structure (Fig. 2a) for detecting N-terminal signal sequences. The training data were acquired by extracting all eukaryotic protein names from SignalP4.0’s dataset and then downloading the corresponding sequences^1^ from UniProt Release 2018-05^10^. The training data contained proteins from three categories, namely, signal peptide-containing proteins (SP), proteins with a transmembrane domain at the N-terminus (TM70) and intracellular proteins (In-cell), from which the first 70 amino acids were extracted for training the CNN model to distinguish the three groups.

It is well known that signal peptides carry a charged N-region, hydrophobic H-region and polar C-region with small uncharged residues at the −1 and −3 positions^2^, and transmembrane domains have a hydrophobic region^11^. To capture motifs underlying the amino acid sequences, which were one-hot coded as 70 × 20 matrices (details in the methods section), the first convolutional layer of the CNN performed channel reduction to compress the 20 dimensions by five kernels. The consequent five feature maps may be interpreted as higher level representations of protein sequence parameters, for instance, hydrophobicity, polarity, charges or their combinations. These feature maps were transformed with rectified linear units (ReLUs) without pooling for this convolutional layer. In the second layer, one-dimensional convolutional kernels ran along the sequence length dimension to detect motif features. Then, we followed a max pooling layer and a ReLU layer, which extracted the maximal feature produced by each one-dimensional kernel. A dense layer then further transformed the learned features to the final softmax layer, providing separate scores for the three classes. Compared to SignalP and DeepSig, the design of the CNN structure made OutCyte-SP a lightweight model that was still efficient at capturing motifs.

The performance comparison with SignalP 4.1^1^, SignalP 5.0^12^ and DeepSig^13^ on three benchmark data sets showed that our CNN model OutCyte-SP achieved comparable or better performance based on the Matthews correlation coefficients (MCCs) of signal peptide identification and micro-averaging MCCs of three-class predictions. As shown by the MCC values (Fig. 2b), OutCyte-SP achieved comparable performance to DeepSig and SignalP 5.0 on benchmark sets from SignalP4.0, DeepSig and SignalP5.0. The performances of OutCyte-SP and DeepSig on the benchmark set of SignalP5.0 were similar but less accurate than that of SignalP5.0. Because both DeepSig and OutCyte-SP are three-class models, the micro-averaged MCCs of their predictions on two benchmark sets were also compared. These comparisons showcased OutCyte-SP’s ability to identify proteins with N-terminal signal peptides, which achieved comparable performance to other state-of-the-art tools.

To further evaluate OutCyte-SP, we applied it to the human proteome and predicted 3,512 signal-peptide-containing proteins, which is similar to the results of previous studies (3,102 proteins by DeepSig, 3,556 proteins by SignalP 4.1 and 3,323 proteins in UniProt Release 2018-05). OutCyte-SP exhibited high agreement with the common resources for signal peptide annotation, and only 90 proteins were unique to OutCyte-SP (Fig. 2c). Moreover, it seems that our CNN model has a more efficient structure than SignalP5.0 and DeepSig for discriminating TM70, In-cell and SP sequences. Therefore, OutCyte-SP appears to be optimal for our integrated computational environment to filter proteins for the cascaded module OutCyte-UPS. It is important to mention that until now, OutCyte-SP has been trained and tested on only eukaryotic proteins.

### OutCyte-UPS: predicting unconventional secretion

Furthermore, we relied on well-defined and representative data sets for developing OutCyte- UPS—a prediction tool for unconventional protein secretion. For proteins utilizing different UPS routes, only a small group of 18 representative proteins was described^7,8^. Therefore, different strategies, such as removing the signal peptide sequences of predicted classically secreted proteins^7^ or considering annotated extracellular proteins^8,14^, have been used to virtually extend the number of candidates for training predictive algorithms. Here, we relied on an in-house data set (157 proteins) of experimentally determined candidate proteins obtained by an integrated secretomic and proteomic approach^15–18^. The list of candidates was shortened to 96 proteins by reducing the number of homologous proteins within the candidate set and removing the 18 previously reported UPS proteins and their homologues (details in the methods section). The negative data for training comprised 345 proteins that were commonly underrepresented in the cellular secretome (details in the methods section) and were therefore less likely to be secreted. For our independent data set for evaluation of OutCyte prediction, the 18 representative UPS proteins were considered as well as 20 proteins from our in-house database, which were highly enriched in the intracellular proteome. As we and others^7,8^ are convinced that the protein sequence properties affect UPS and thereby the physicochemical properties of the involved amino acids, 61 features were considered (Table S1) as being potentially informative for UPS.

**Figure 3:**
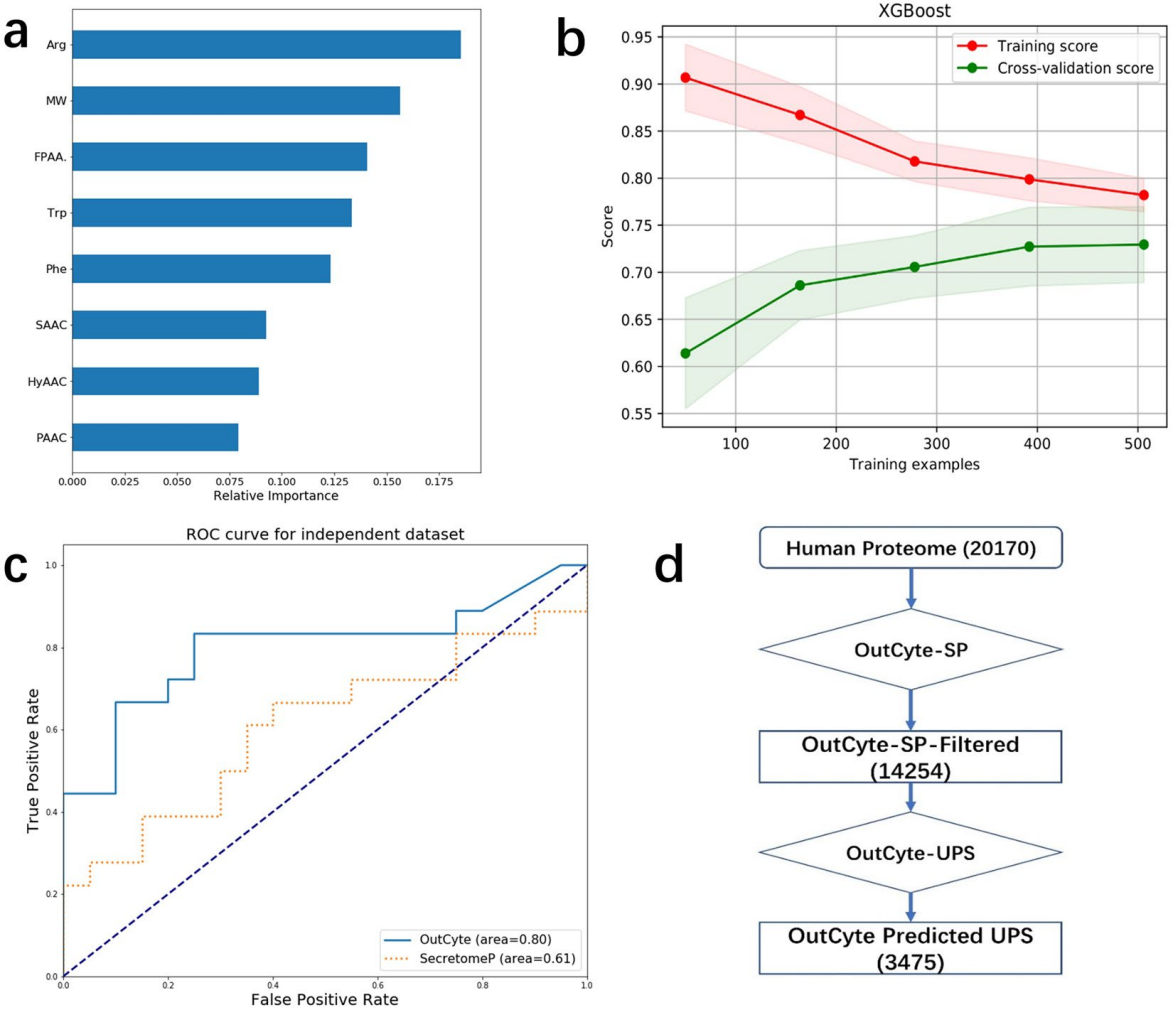
Prediction of unconventional protein secretion by OutCyte-UPS. (**a**) Eight features were identified to be important for the classification of unconventionally secreted proteins. Important features include a high frequency of arginine residues and positively charged amino acids, which have already been previously associated with the membrane transition of proteins. (**b**) Cross-validated training curve for XGBoost-based OutCyte-UPS. (**c**) An independent data set containing experimentally verified UPS proteins as well as the top 20 intracellular proteins from experimental data was used for performance comparison. Here, OutCyte-UPS showed improved performance compared to SecretomeP. (**d**) The OutCyte pipeline was applied to all 20170 proteins from the human proteome: OutCyte-SP classified 6077 proteins as containing either an N-terminal signal peptide or a transmembrane domain. The remaining 14,254 proteins were then used for OutCyte-UPS prediction of unconventional secreted proteins. Finally, 3,475 human proteins were predicted to be unconventionally secreted.

To build models from the small and imbalanced data sets, we first performed an effective feature selection and finally kept 8 features (Fig. 3a) and oversampled the positive set to balance the negative set (details in the methods section). Then, the model based on XGBoost^19^ was trained by the same nested cross-validation scheme as OutCyte-SP, achieving a cross-validation score of 0.73. When applied to the benchmark dataset, OutCyte-UPS correctly classified 14 out of 18 UPS proteins and achieved an AUC of 0.80 by ROC analysis (Fig. 3b, Table S2). The misclassified proteins were H4-Human, FGF2-Human, THTR-Human and HMGB1_Human. In contrast, SecretomeP achieved an AUC of 0.61 and misclassified 9 UPS proteins.

Next, we were interested in evaluating individual feature contributions for predictions using OutCyte-UPS. Here, the feature ranking for single protein predictions is consistent with the result from the independent data set: in addition to the physicochemical features at the C-terminus, the molecular weight and positively charged amino acids, the frequency of arginine within the protein sequence contributes significantly (Figs. 3a and S1, S2). Notably, the role of arginine in protein transport is well established. In plants, bacteria and archaea, arginine plays a role in the signal motif of the *twin-arginine translocation* (Tat) system, which contains a characteristic twin-arginine motif within the N-terminal signal peptide. Moreover, several computational and experimental studies^20,21,22^ have reported that arginine has a strong mechanistic role in arginine-rich cell-penetrating peptides (CPPd), enhancing the ability of these peptides to transverse the cell membranes of many mammalian cells. The significance of arginine content in a protein shown in our study may indicate its important role in promoting UPS in at least a subgroup of the secretome. By manual inspection, we revealed that FGF2-Human and H4-Human, misclassified by OutCyte-UPS, exhibited higher frequencies of positively charged amino acids and arginine than all other samples in both independent and training positive data sets (Figs. S3 and S4), which may have contributed to their misclassification.

### Annotating the human proteome

Alternative secretion routes were long neglected, and secreted proteins without a signal peptide were commonly considered contaminants. Therefore, we were interested in predicting the number of classic secreted proteins as well as UPS in the human proteome. Of the reviewed human proteome comprising 20,170 proteins, we predicted 1,829 proteins with a signal peptide for classic secretion by OutCyte-SP. This number is in the range obtained by other prediction tools/databases: 1,836 proteins (SignalP 4.1), 1,693 proteins (DeepSig), and 1,999 proteins (UniProt) (Fig. S5). From the 14,245 proteins remaining after OutCyte-SP filtering, we predicted 3,475 UPS proteins in the human secretome by OutCyte-UPS (17.1% of the reviewed human proteins) (Fig. 3d). In contrast, SecretomeP identified 6,688 UPS proteins, accounting for roughly one-third of the reviewed human proteome (Figs. 3d and S6, S7). However, as the exact number of UPS candidates is still unknown, we cannot exclude a bias towards a specific secretory pathway overrepresented in our secretome data sets.

## Conclusion

With our presented experimental data-driven approach, we built OutCyte for predicting potential unconventional protein secretion. The first part, OutCyte-SP, proved the ability of this system to efficiently and accurately identify N-terminal signals, such as signal peptides and transmembrane domains. The cascaded OutCyte-UPS was trained on our experimental data and outperformed SecretomeP on the currently known unconventionally secreted proteins. We are convinced that this system will provide new perspectives on the unknown processes of UPS and that we have laid the foundation for improved prediction of UPS using experimentally verified UPS proteins in the future.

## Methods

### Training and benchmark datasets for OutCyte-SP

OutCyte-SP was trained on eukaryotic proteins extracted from SignalP4.0’s dataset. To obtain the up-to-date sequences, the eukaryotic protein names were used to retrieve sequences from UniProt Release 2018-5. In the training set, 1361 proteins possessed a signal peptide with experimental evidence annotated in UniProt; 913 proteins with transmembrane domains annotated at the first 70 amino acids were extracted; and 4491 proteins from the nucleus or cytoplasm were kept to represent proteins without N-terminal signals.

To benchmark OutCyte-SP with SignalP 4.0, SignalP 5.0 and DeepSig, we tested all four models on three benchmark datasets (SignalP 4.0 benchmark set, SignalP5.0 benchmark set and DeepSig benchmark set; due to the overlap between the SignalP5.0 training set and SignalP 4.0 benchmark set, two MCC values were not included in Fig. 2a). The detailed statistics of the benchmark sets are shown in Table S3.

The protein identities were extracted from datasets of SignalP 4.0 and SignalP 5.0 and used to retrieve the sequences from UniProt Release 2018-5.

### Datasets for OutCyte-UPS

In a recent approach, we described the comparison of abundances of secreted and cellular proteins as a valuable tool to select proteins that are enriched in the secretome and therefore probably secreted^15^. Using this approach in ten different experimental settings, including mouse, rat, and human cell types^16,17^, we developed a database containing proteins showing a high likelihood to be secreted in the respective systems (called secreted proteins in this chapter) as well as those showing very high likelihood to not be secreted (called cellular proteins in this chapter). The proteins that were secreted in at least three different experiments and did not contain a signal peptide and/or transmembrane domain (UniProt Annotation release May 2018) were selected as the training set for OutCyte-UPS. Notably, the computational annotations of signal peptides and transmembrane domains in UniProt were also included in our data processing. Potential false positives/negatives may be present in the annotations due to protein isoforms, false prediction and so on. However, the high accuracy and confidence of the tools^12^ used for predicting signal peptides could keep noise level low in our processed data. Grube *et al*.^15^ experimentally validated the detection of secretome-enriched proteins, e.g., by inhibiting classic secretion by Brefeldin A, 95% of proteins whose secretion was inhibited by Brefeldin A indeed had a signal peptide annotated in UniProtKB. Therefore, the noise was kept at a low level in our processed data. A certain level of noise in training data is usually expected when developing a machine learning model^23^. Since the same gene might encode proteins with different names in different organisms, the presence of each protein in different experiments was counted in terms of the encoding gene. If the same gene was present in multiple organisms with different protein names, the human protein homologue was kept in the positive data. In total, we obtained 157 unconventional secretory proteins. We further cleaned up the data by removing proteins sharing sequence identities above 30% with proteins in the independent data, which consisted of 18 positive proteins from the literature and 20 negative proteins selected from our experiments (explained later). The final UPS training data set contained 96 proteins. Similarly, the intracellular proteins were prepared as a negative data set for training. The proteins that were highly abundant in the cellular proteome but rare in the secretome were kept as candidates. Among these candidates, proteins with signal peptides or transmembrane domains as well as proteins sharing a sequence identity higher than 30% with other used sequences were removed.

To generate reliable independent negative data for evaluating OutCyte-UPS, the top 0.5% of the proteins enriched in cell lysates and underrepresented in secretomes were extracted from our database. Because OutCyte-UPS was focused on identifying UPS from proteins without both transmembrane domains and signal peptides, the extracted proteins with either transmembrane domains or signal peptides were excluded, resulting in 20 proteins as negative evaluation data. The sequence length distributions of different datasets are plotted in Fig. S8, which shows that the medians and means were not biased towards either positive or negative training sets.

### Preparation of human proteome sequences

To scan for unconventional secretory and signal peptides containing proteins in the human proteome, we extracted the reviewed entries in UniProtKB Release 2018-05 for the human proteome of 20,170 proteins, which is divided into four categories: proteins with signal peptides and transmembrane domains, proteins with signal peptides but not transmembrane domains, proteins without signal peptides and transmembrane domains and proteins with transmembrane domains but not signal peptides. The statistics of this categorization are summarized in Fig. S9.

### Model metrics

Multiple metrics were calculated for the model benchmarks. This section explicitly defines all the used metrics, which are abbreviated as follows: TP for the number of true positives, TN for true negatives, FP for false positives and FN for false negatives.

*Accuracy (ACC)*

$$\begin{array}{rcl}ACC & = & \frac{TP+TN}{(FN+TP+TN+FP)}\\ ACC & = & \frac{TP+TN}{(FN+TP+TN+FP)}\end{array}$$

*Sensitivity or true positive rate (TPR)*

$$TPR=\frac{TP}{FN+TP}$$

*Specificity or true negative rate (TNR)*

$$TNR=\frac{TN}{TN+FP}$$

*Matthews correlation coefficients*

$$MCC=\frac{TP\times TN-FP\times FN}{\sqrt{(TP+FP)(TP+FN)(TN+FP)(TN+FN)}}$$

*Micro-averaging for three classes, namely, 0, 1, and 2:*

$$\begin{array}{rcl}TP & = & T{P}_{0}+T{P}_{1}+T{P}_{2}\\ FN & = & F{N}_{0}+F{N}_{1}+F{N}_{2}\\ TN & = & T{N}_{0}+T{N}_{1}+T{N}_{2}\\ FP & = & F{P}_{0}+F{P}_{1}+F{P}_{2}\end{array}$$

### One-hot coding representations of amino acid sequences

Twenty standard amino acids were considered in this work. After sorting the amino acid letters in alphabetic order, each amino acid letter was encoded by a 20-dimensional vector with its position in the alphabetic order set to 1 and those of the rest to 0. Because each encoded vector has only one entry set to 1, it is called a one-hot coding scheme. Therefore, a protein sequence with L amino acids is represented by an L x 20 matrix.

### Convolutional neural network

Convolutional neural networks (CNNs)^24^ have the properties of translational invariance and local spatial coherence due to the convolutions between the input matrix and filtering kernels, where the kernels parameterized by weights are expected to extract features from the input by tuning weight values during learning. CNN models are suitable for learning patterns, e.g., the signal peptide motif and transmembrane domain in amino acid sequences but with varying locations on the sequences.

CNN structures typically have a number of convolution layers for extracting features of different levels from the inputs, and each layer typically consists of operations of convolution, pooling and transformation for its input. The operations have different variants for different tasks. In this work, standard convolution, max pooling, and rectified linear units (ReLus) were used. Max pooling means that for a fixed window from a convolved feature map, the maximal value is used for representing the window. By max pooling, the dimensions of feature maps are reduced (down-sampling), and the best feature of each window is kept. For example, max pooling can sharpen the edges of blurry items in an image^25^. The same idea was applied to learn features from sequences. The ReLu transformation is defined as $f(x)=max(0,x)$, which provides a simple nonlinear transformation for accelerating the training of neural networks.

The CNN model was implemented and optimized in Theano^26^.

### Training the OutCyte-SP CNN model with nested cross-validation

Cross-validation is usually used for optimizing machine learning models; for instance, the *k*-fold cross-validation divides the entire dataset into *k* partitions, where *k-1* partitions are used for training and validating the model, while one partition is left out for testing the model performance. As discussed in the paper for SignalP 4.0^1^, the standard k-fold procedure is sufficient if the model is blinded to the test data during the training procedure, i.e., the test data should not be used for either hyperparameter tuning or model selection. To overcome this problem, nested cross-validation is applied to tune the models, which further performs inner *n*-fold cross-validation on the k-1 partitions. To benchmark with SignalP 4.1 and DeepSig, we used the same cross-validation setup to tune hyperparameters and select models: one partition of a 5-fold was kept out, and an inner 4-fold cross-validation on the remaining four partitions was performed to optimize the CNN hyperparameters, for example, the learning rate and the mini-batch size, and the CNN structures, for example, the number of convolution layers, number of kernels, and kernel sizes. After the nested cross-validation procedure, 20 CNN models with the same structures trained with the same hyperparameters but with different inner training partitions were obtained for constructing the final ensembled model for application.

### Feature generation and selection for OutCyte-UPS

For classifying UPS, given the dataset of 96 positive and 345 negative examples, proteins need to be represented by informative characteristics in terms of secreted proteins, unlike the prediction of signal peptides, where one-hot-coded raw sequences can be directly used as input features for convolutional neural networks for extracting the clear motif, and the dataset size is much larger than the UPS dataset. Moreover, as reported by Bendtsen *et al*.^7^, we did not find any clear motif in the 18 reported unconventional secretory proteins.

To generally characterize the proteins, amino acid compositions represent the individual amino acid frequencies over the entire sequence. For example, the frequency of amino acid *i* (*AA*_*i*_) is calculated as

$$A{A}_{i}=\frac{Number\,of\,A{A}_{i}}{Total\,sequence\,length}.$$

Many studies have reported that the molecular weights of proteins influence protein secretion. We also plotted histograms of human classic secretory proteins and the human proteome in Fig. S10, which shows that relatively small proteins are favoured for secretion. To this end, molecular weights were calculated by the *molecular_weight()* function in Biopython^27^ as protein features.

Physicochemical features, such as hydrophobicity, polarity, and positively or negatively charged residues, are widely believed to play a critical role in protein secretion. Small amino acids are also considered for feature generation because they also affect protein functions^28^, and we hypothesize that their levels in a protein sequence might influence the secretion of proteins. To characterize the positional physicochemical features of protein sequences, the frequencies of different amino acid groups (hydrophobic, polar, positively charged, negatively charged and small) were calculated for segments as shown in Fig. S11.

We did not account for protein folding structures within our list of features, as even for classic secretion, in most cases, the exact process of folding and maturation during the ER-Golgi passage is not clearly defined. In bacteria, the two major modes of protein secretion follow either the Sec or Tat (*twin-arginine translocation*) pathway. Proteins are folded after secretion when secretion is mediated by the Sec machinery, whereas the Tat machinery carries folded proteins to the outside of bacteria. Both the Sec and Tat pathways have also been found in eukaryotes^29^.

In total, 61 features have been generated for individual proteins, which are summarized in Table S1. An exploratory data analysis for all the features of the UPS dataset shows the correlation of the features (Fig. S12). Due to the limited size of the available UPS dataset, we performed an extensive feature selection to keep only the most representative features. The feature importance ranking shown in Fig. S1 was obtained by averaging 500 single rankings using the Random Forest classifier in Scikit-learn 0.19 on the merged dataset from both the training data and independent data. Notably, the real population of UPS in even the human proteome remains unknown; therefore, both our 96 positive examples and 18 reported proteins are merely samples from the population and are highly likely to suffer from sample selection bias. Due to the bias, many features could be identified as drifting features that have a strong discriminative power for the training dataset and independent dataset. The ranking of drifting features is shown in Fig. S2. Using features that are important and exhibit less drift, we further performed a best-one search^30^ to keep the top feature combinations, which resulted in our final features.

The subsequent feature selection was based on feature importance ranking and feature drifting analysis. To avoid bias due to different sample sources, we considered both training and test data from the same distribution^31,32^. Furthermore, we selected the features that were top ranked in terms of importance but low ranked in terms of drifting. Finally, the features considered by OutCyte-UPS include the molecular weight; frequencies of small, hydrophobic and positively charged residues in the C-terminus; frequency of positively charged residues over the entire sequence; and frequencies of tryptophan, phenylalanine and arginine.

Another common challenge in machine learning tasks is the unbalanced training dataset. We have 345 negative examples but only 96 positive samples. To handle the imbalanced dataset, we chose to oversample the minority rather than down-sampling the majority due to the small datasets. Repeated oversampling, synthetic minority oversampling technique (SMOTE)^33^ and adaptive synthetic oversampling approaches (ADASYN)^34^ were applied to oversample the positive dataset and to balance it with negative datasets. Repeated oversampling is simply duplicating positive examples to match the number of negative examples. Both SMOTE and ADASYN oversampled the positives by generating a new synthetic example with the assumption that the individual feature values are continuous such that similar features can be generated with values next to a given example.

### Model training and selection for OutCyte-UPS

Due to the small UPS dataset, logistic regression, random forest and gradient boosting trees have been extensively tested and compared. In terms of binary classification with classes 0 and 1, logistic regression performs a sigmoidal transform of linear combination of input features to values falling in the range of [0, 1], which can be interpreted as the probabilities of being in class 1. Random forest and gradient boosting are both tree-based ensemble learning algorithms. Trees in random forest are parallelly grown and are not correlated to each other. Each tree is trained on bootstrapped samples of the original training data, and the output of a random forest is obtained by averaging outputs from all trees^35^. Gradient boosting trees grow CARTs (Classification and Regression Trees) sequentially to fit each tree to the current residue given by its preceding tree; in other words, it additively ensembles weaker learners (CARTs) in a sequential manner to obtain powerful models^19^.

As we have an independent dataset for evaluating the final model predictions, a routine of nested cross-validation with a grid search of hyperparameters was used for training models to make full use of the limited dataset and avoid information leakage from the training data to test data. Similar to the training OutCyte-SP, the cross-validation led to 20 runs for each hyperparameter setting. Then, averaged metrics for training and testing were obtained from 20 runs. The averaged MCC metrics were used for selecting models.

Probability calibration for the tree-based models is often needed because we intend to not only predict the class for the given data point but also obtain a well-calibrated probability as the confidence of being in a certain class. For example, it is difficult for methods such as random forest to make predictions with scores of 0 or 1 because the variance from individual trees drag the actual predicted scores from zero or one as they should be^36^. We used the parametric “sigmoid” and nonparametric “isotonic” methods implemented in Scikit-Learn 0.19 to calibrate the final prediction scores.

## Supplementary information

OutCyte: a novel tool for predicting unconventional protein secretionSupplementary information

## Data Availability

The code and datasets generated during and/or analysed during the current study are available from the corresponding author on reasonable request.
