# Supplementary material for: OutCyte: a novel tool for predicting unconventional protein secretion
Source: Sci Rep. 2019 Dec 19;9:19448. doi: 10.1038/s41598-019-55351-z (PMC6923414; doi:10.1038/s41598-019-55351-z)
Supplement: Supplementary file 1 — OutCyte: a novel tool for predicting unconventional protein secretionSupplementary information [file 41598_2019_55351_MOESM1_ESM.pdf]

OutCyte: a novel tool for predicting unconventional protein secretion- Supplementary information

Linlin Zhao[1,2], Gereon Poschmann[1], Daniel Waldera-Lupa[1], Nima Rafiee[2], Markus Kollmann[2], Kai Stühler[1,3 *]

[1]Institute of Molecular Medicine, Medical Faculty, Heinrich-Heine-University, Düsseldorf, Germany

[2]Mathematical Modelling of Biological Systems, Heinrich-Heine-University, Düsseldorf, Germany

[3] Molecular Proteomics Laboratory, BMFZ, Heinrich-Heine-University, Düsseldorf, Germany

*Correspondence: kai.stuehler@hhu.de (K. Stühler)

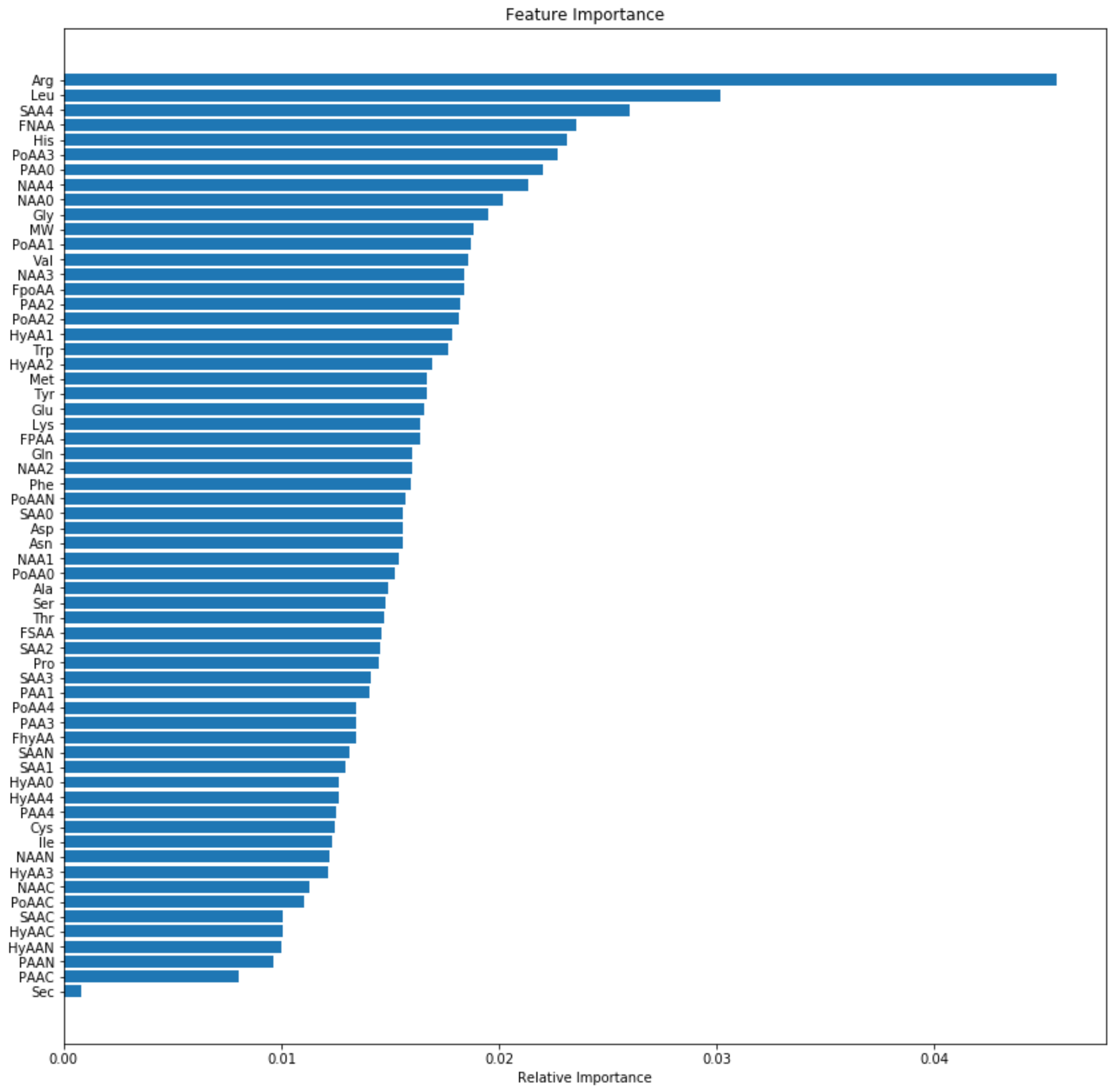

Fig. S1 Feature importance ranking

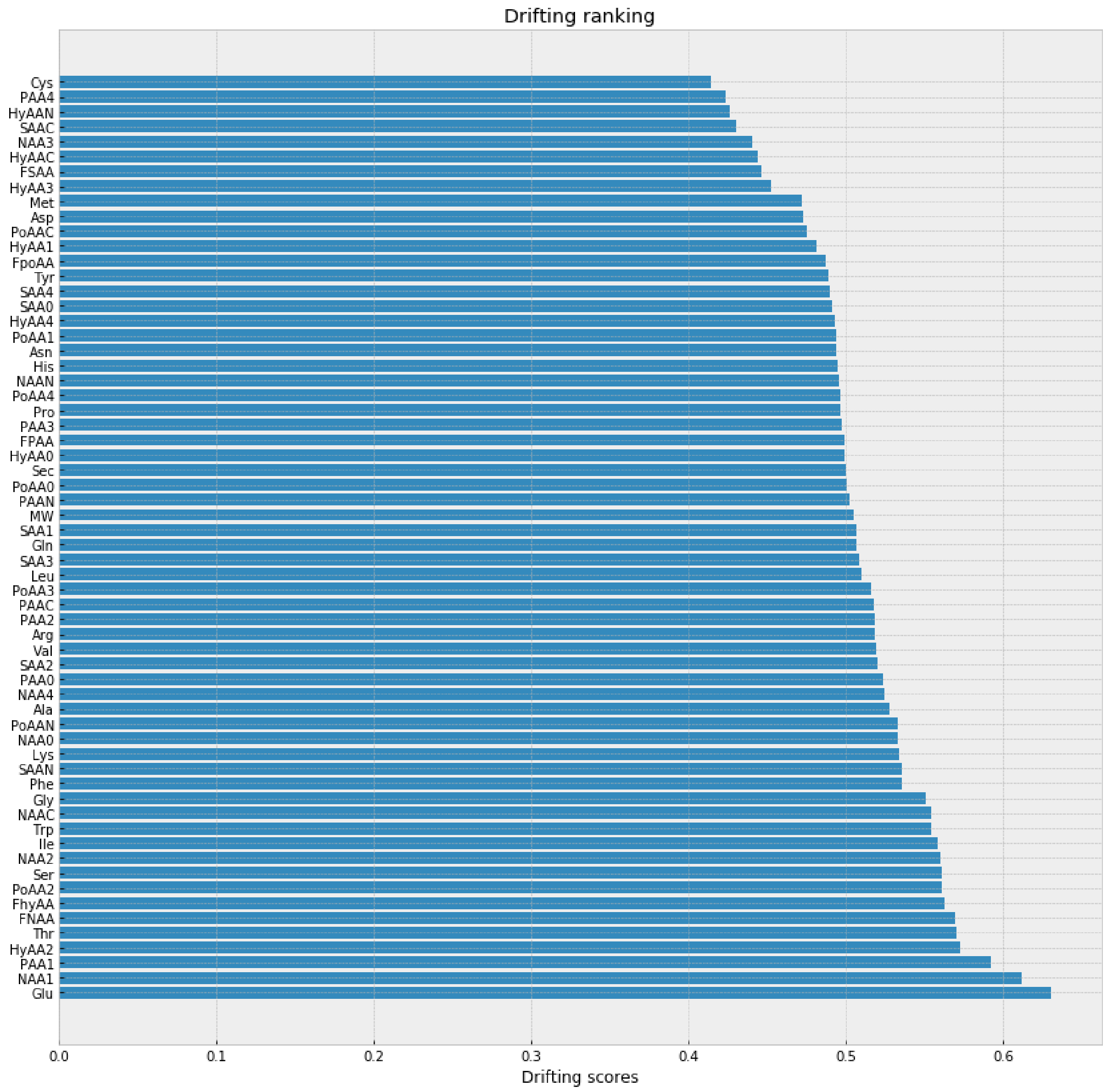

Fig. S2 Drifting ranking for features

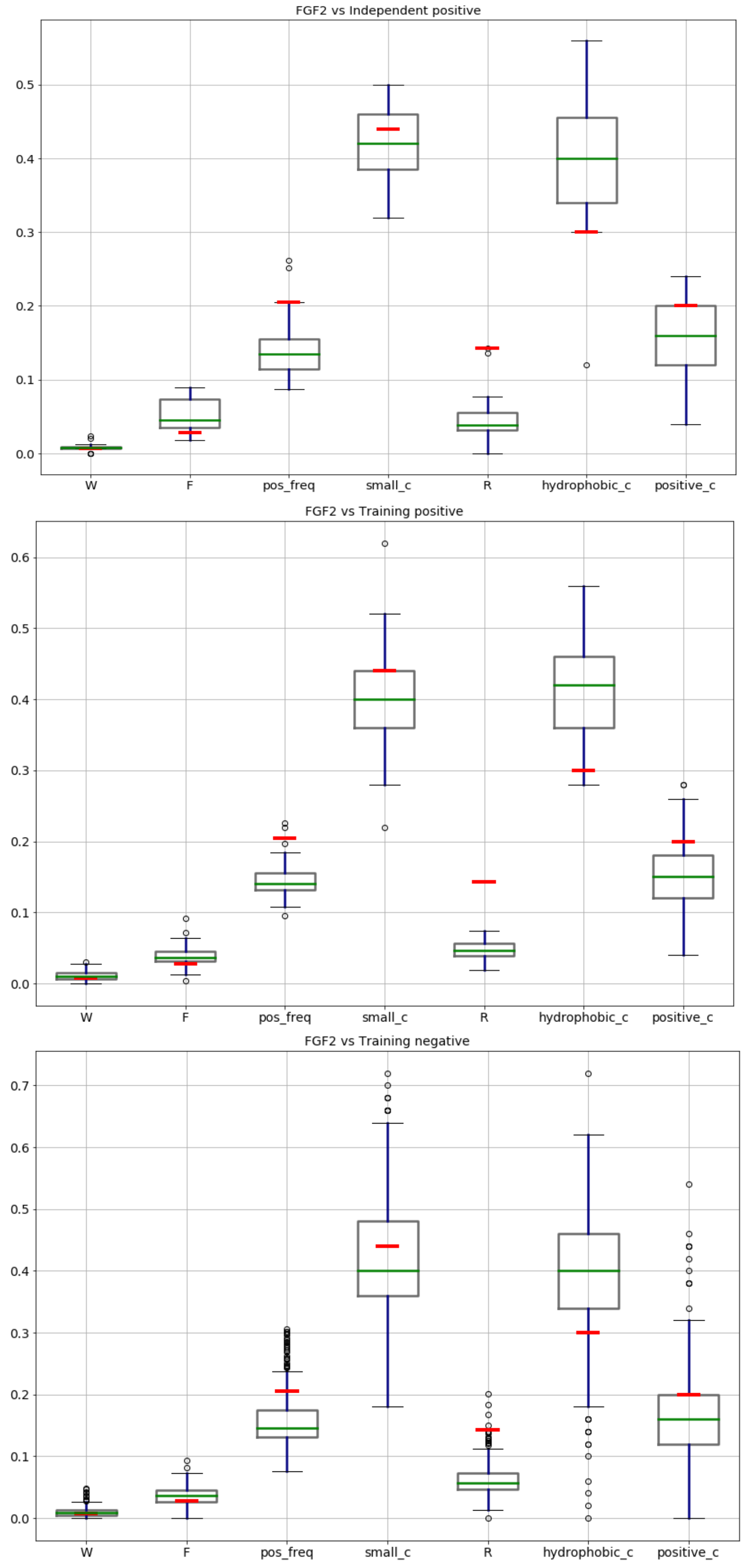

Fig. S3 The FGF2-Human's features (the red horizontal line) compare to the boxplot of features in different data sets. The y-axes stand for the values of the features on x-axes.

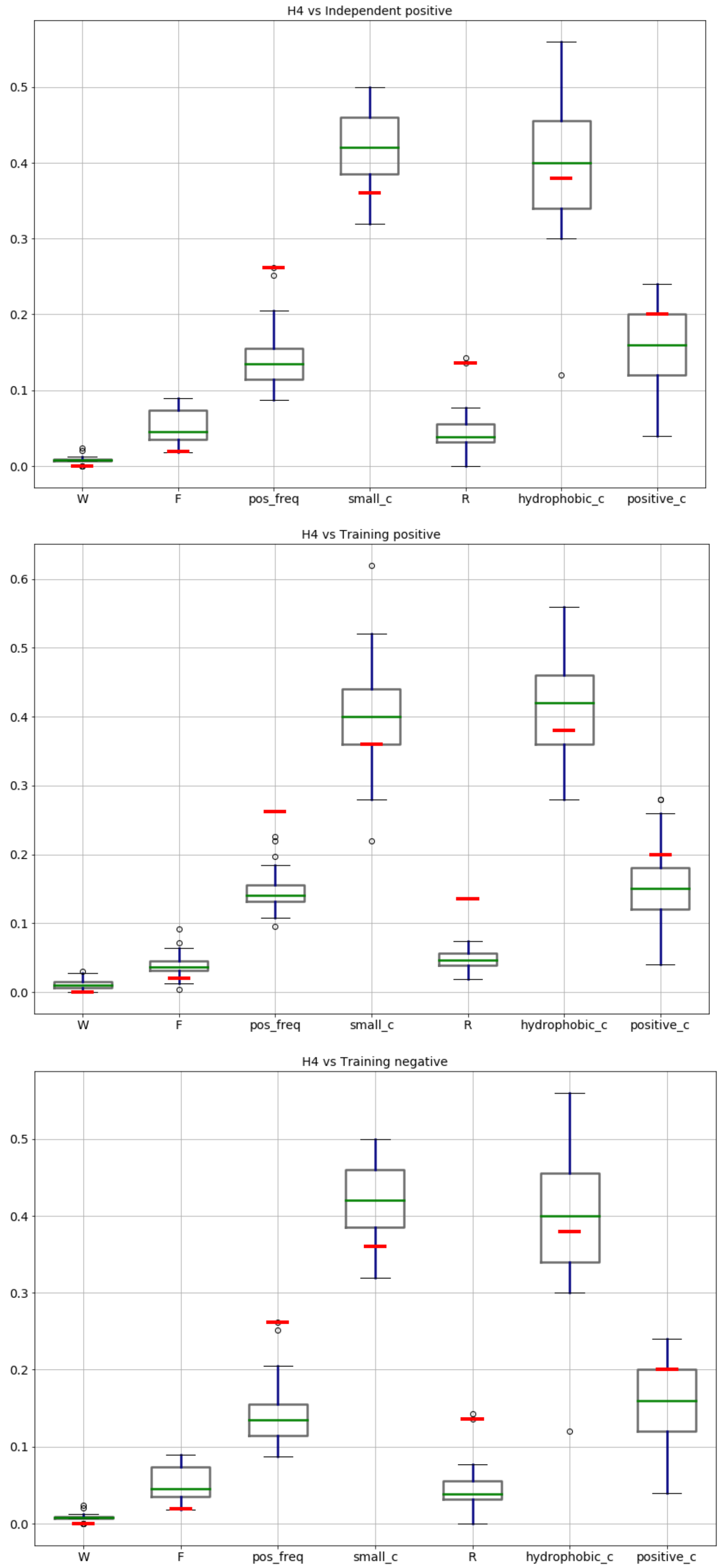

Fig. S4 The H4-Human's features (the red horizontal line) compare to the boxplot of features in different data sets

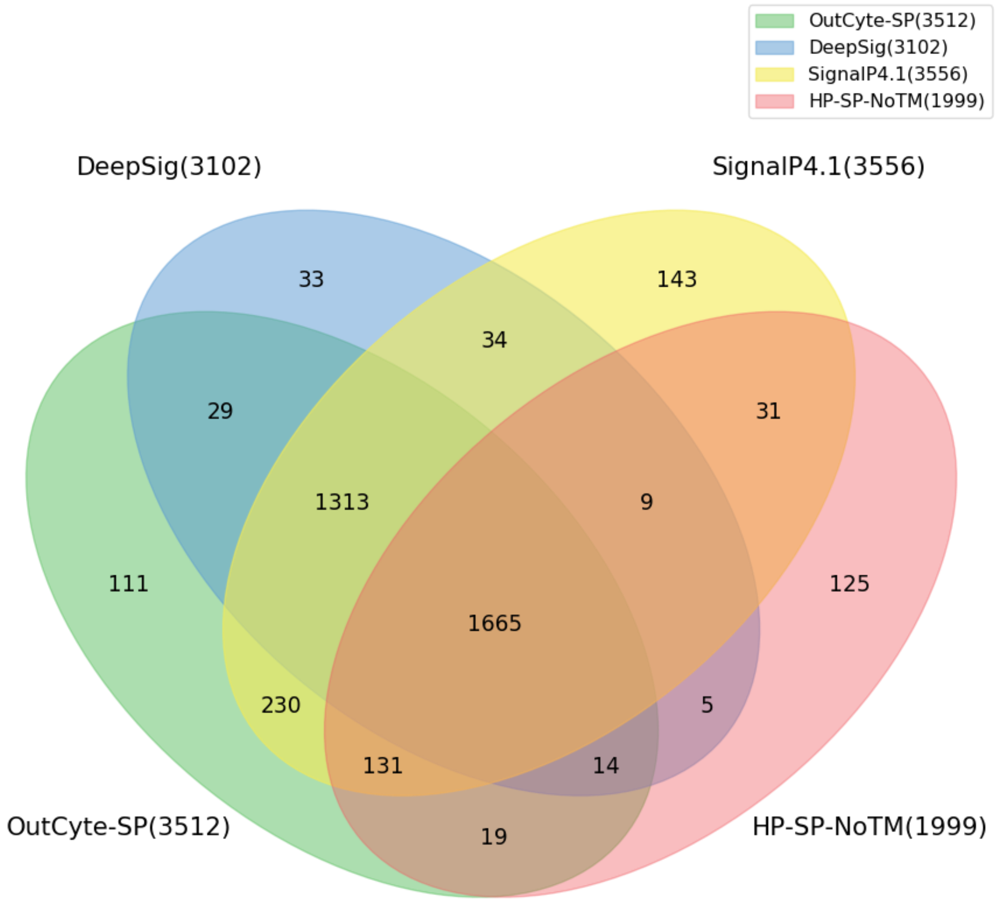

Fig. S5 Predictions of signal peptides within human proteins using different tools and databases (HP-SP-NoTM = signal peptide annotated in the UniProt database)..

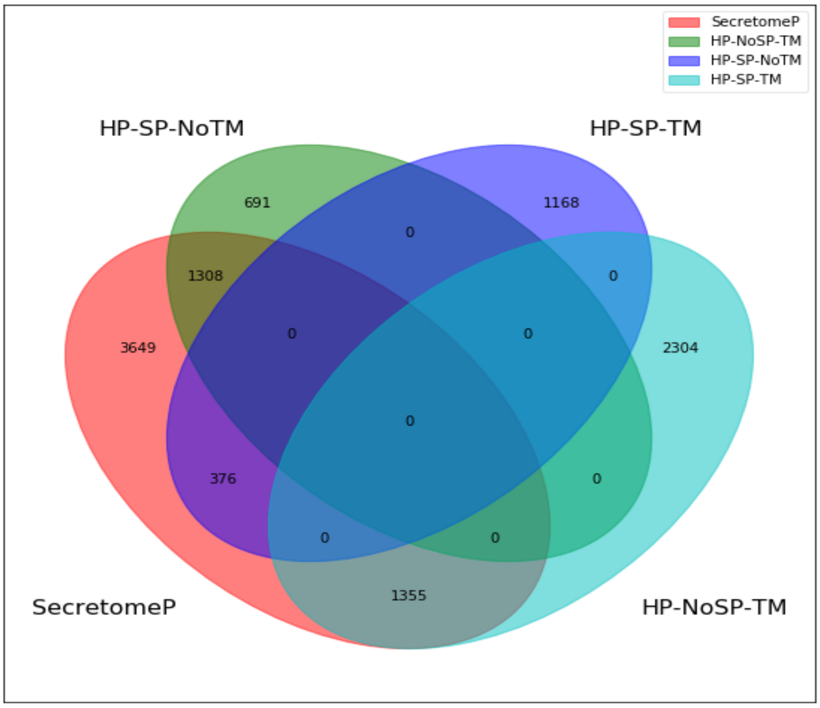

Fig. S6. The Venn diagram for SecretomeP prediction's intersection with three human proteome subgroups.

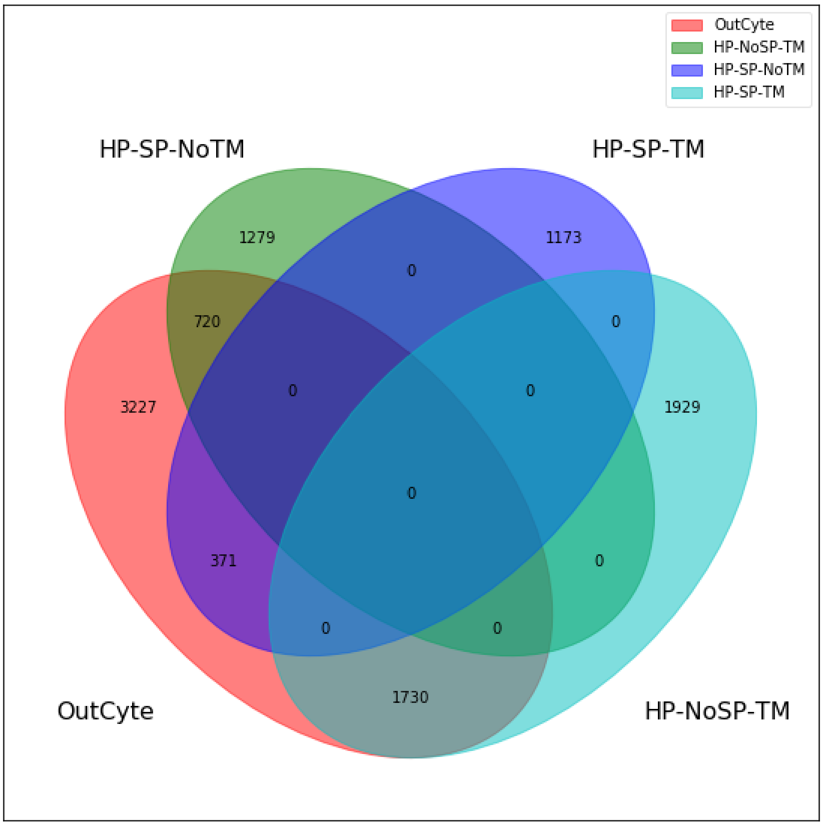

Fig. S7 The Venn diagram for OutCyte prediction's intersection with three human proteome subgroups.

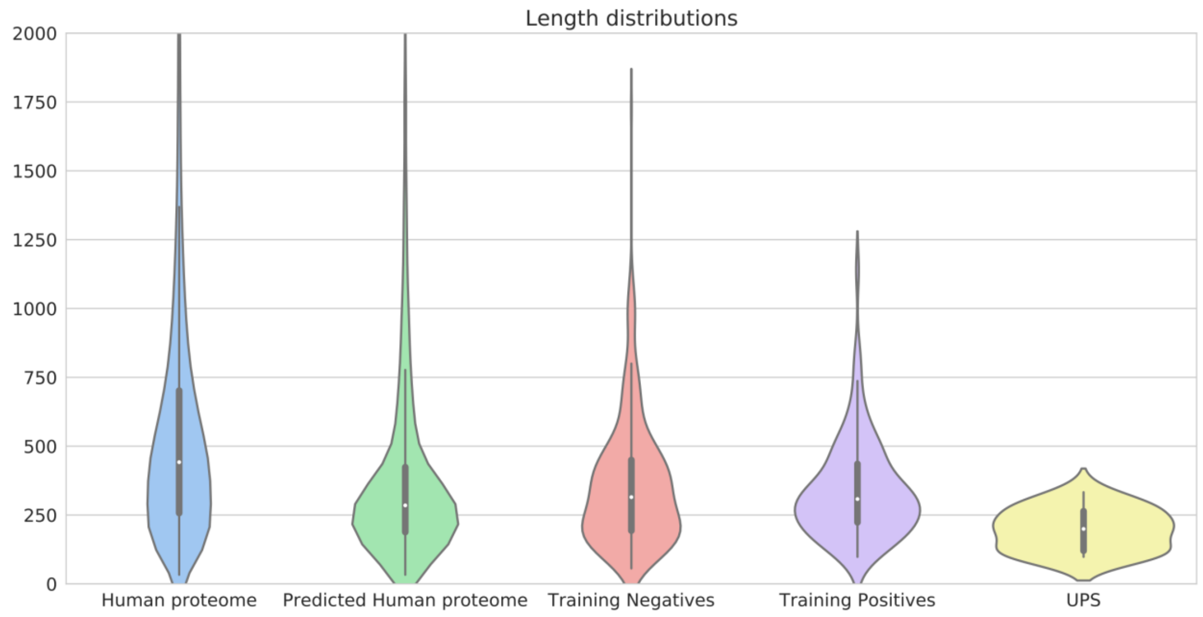

Fig. S8. Length distributions for datasets related to OutCyte-UPS

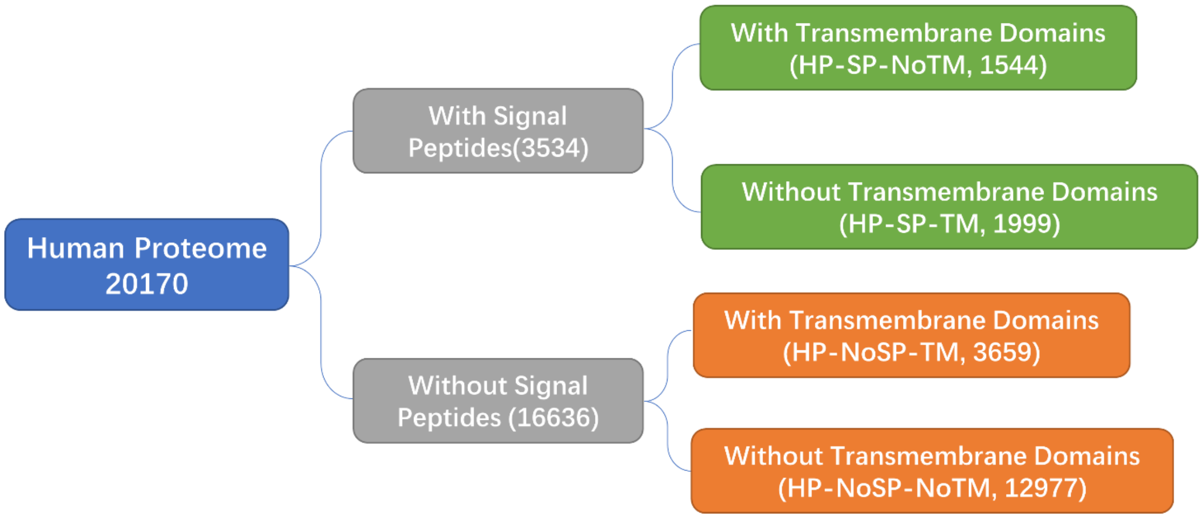

Fig. S9. Human proteome subgroups.

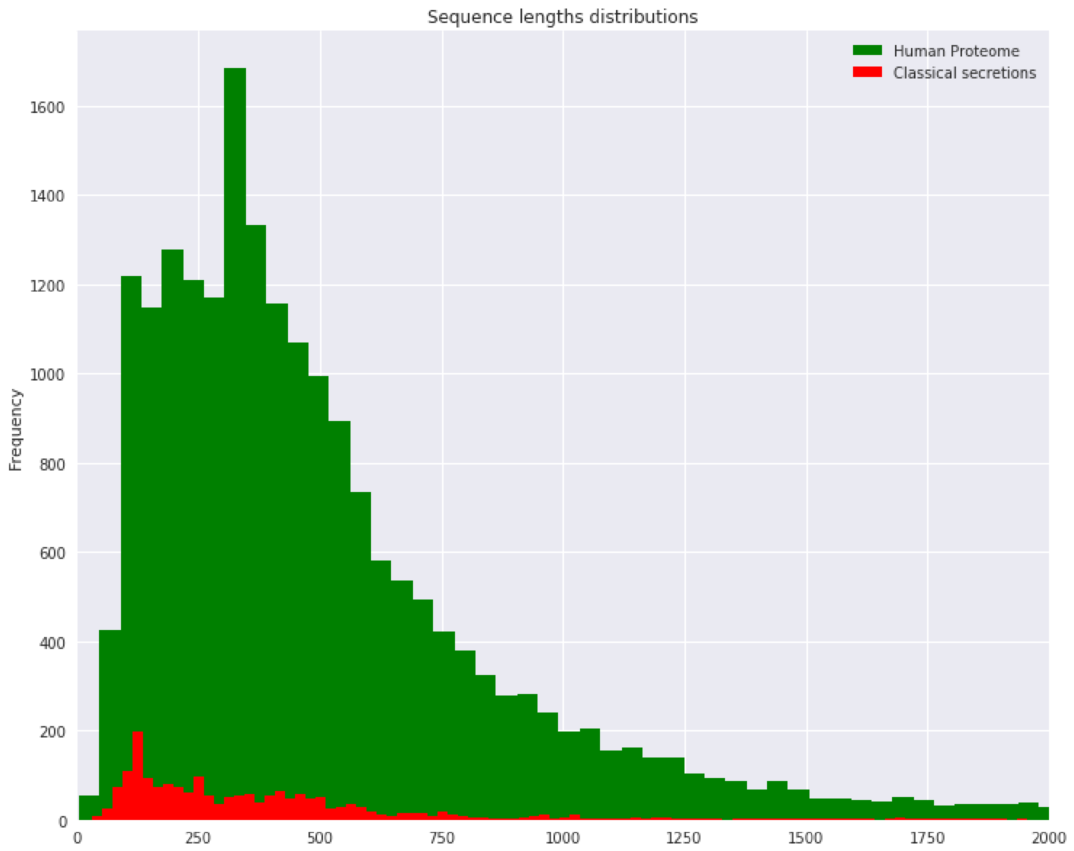

Fig. S10. The length distribution of human classical secretory proteins and human proteome, which showed the favor of smaller molecular in terms of secretion.

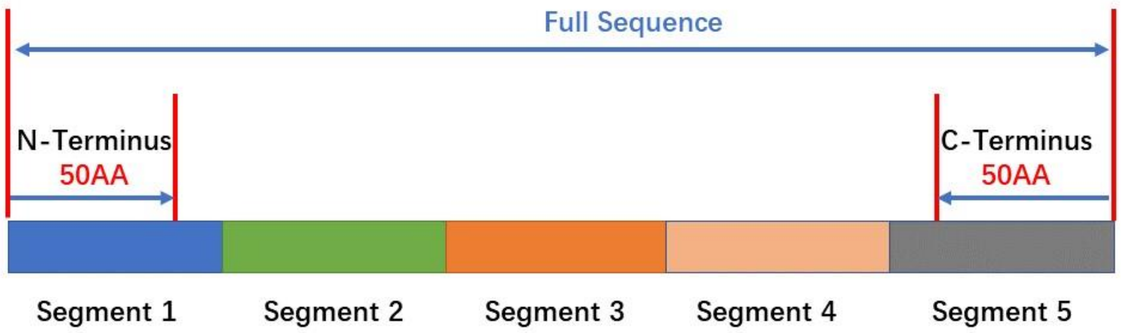

Fig. S11 Segmentations of sequences for generating positional physicochemical features

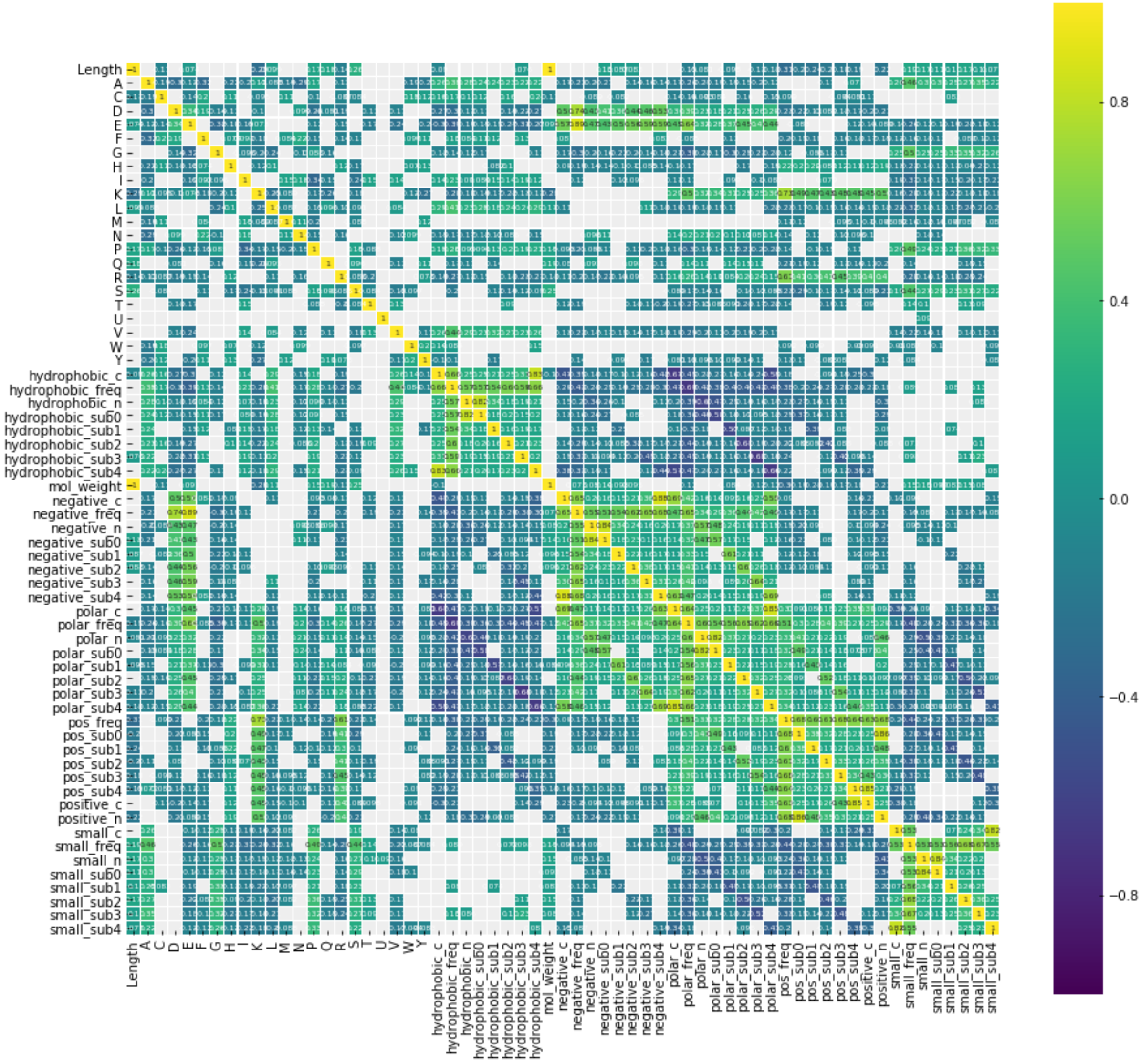

Fig. S12. The correlations of 61 features generated for building OutCyte-UPS.

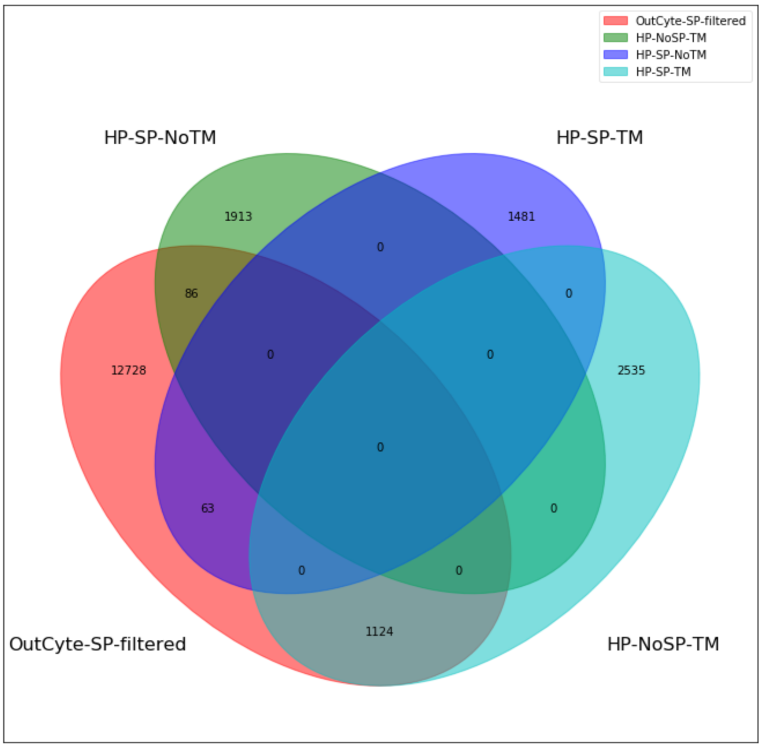

Fig. S13. The proteins without an N-terminal signal predicted by OutCyte-SP intersect with other human proteome subgroups. It shows the ability of OutCyte-SP to filter away proteins with N-terminal signals.

Table S1 List of 61 features for representing sequences

Size	Feature Names	Abbreviations
1	Molecular Weights	MW
20	Amino acid frequencies of entire sequence	Met, Cys, Trp, Phe …
3	Small amino acid frequencies of entire sequence, N- and C-terminus	FSAA, SAAN, SAAC
3	Positively charged amino acid frequencies of entire sequence, N- and C-terminus	FPAA, PAAN, PAAC
3	Negatively charged amino acid frequencies of entire sequence, N- and C-terminus	FNAA, NAAN, NAAC
3	Polar amino acid frequencies of entire sequence, N- and C-terminus	FPoAA, PoAAN, PoAAC
3	Hydrophobic amino acid frequencies of entire sequence, N- and C-terminus	FHyAA, HyAAN, HyAAC
5	Positively charged amino acid frequencies of 5 sequence segments	PAA1, PAA2, …, PAA5
5	Negatively charged amino acid frequencies of 5 sequence segments	NAA1, NAA2, …, NAA5
5	Polar amino acid frequencies of 5 sequence segments	PoAA1, PoAA2, …, PoAA5
5	Hydrophobic amino acid frequencies of 5 sequence segments	HyAA1, HyAA2, …, HyAA5
5	Small amino acid frequencies of 5 sequence segments	SAA1, SAA2, …, SAA5

Table S2 Predictions on known UPS

Protein	UniProt ID	OutCyte-UPS	SecretomeP	SRTpred
**FGF1-Human**	P05230	0.616(+)	0.847(+)	-0.81(-)
**FGF2-Human**	P09038	0.066(-)	0.239(-)	0.8(+)
**IL1B- Human**	P01584	0.598(+)	0.610(+)	0.96(+)
**IL1A- Human**	P01583	0.615(+)	0.551(-)	-0.2(-)
**LEG3-Human**	P17931	0.618(+)	0.770(+)	-1.16(-)
**MIF-Human**	P14174	0.584(+)	0.776(+)	-0.91(-)
**S10A4-Human**	P26447	0.614(+)	0.724(+)	-0.55(-)
**GSTP1-Human**	P09211	0.598(+)	0.545(-)	-0.7(-)
**PRDX1-Human**	Q06830	0.618(+)	0.528(-)	-0.94(-)
**IL18-Human**	Q14116	0.614(+)	0.634(+)	-1(-)
**H4-Human**	P62805	0.065(-)	0.408(-)	-1.12(-)
**S10A2-Human**	P29034	0.614(+)	0.324(-)	-0.48(-)
**LEG1-Human**	P09382	0.598(+)	0.345(-)	-0.62(-)
**THIO-Human**	P10599	0.617(+)	0.370(-)	0.71(+)
**CNTF-Human**	P26441	0.571(+)	0.653(+)	0.02(+)
**HME2-Human**	P19622	0.525(+)	0.727(+)	-1.39(-)
**THTR-Human**	Q16762	0.066(-)	0.616(+)	-1.2(-)
**HMGB1-Human**	P09429	0.499(-)	0.068(-)	-1.2(-)

Table S3 Statistics of datasets for training and evaluating OutCyte-SP

	**SP**	**TM**	**N/C**	**Globular**
**Training**	1361	913	4491	
**Evaluation-SignalP4**	609	939	1001	
**Evaluation-DeepSig**	46	323	688	
**Evaluation-SignalP5**	211			7248

Table S4 Signal peptide prediction benchmarks

	**OutCyte-SP**	**DeepSig**	**SignalP4.0**	**UniProt**
**OutCyte-SP**	3512	3021	3339	2983
**DeepSig**		3102	3021	2739
**SignalP 4.0**			3556	3009
**UniProt**				3323
